# Supplementary material for: Exploratory and confirmatory factor analyses identify three structural dimensions for measuring physical function in community-dwelling older adults
Source: PeerJ. 2023 Apr 21;11:e15182. doi: 10.7717/peerj.15182 (PMC10355189; doi:10.7717/peerj.15182)
Supplement: Supplemental Information 3 [file peerj-11-15182-s003.docx]

**Raw data codebook**

The “1” in the "Gender" represent male; The “2” in the "Gender" represent female.

**Abbreviations:**

TUG, timed up and go

UWS, usual walking speed

MWS, maximal walking speed

CST, 30-s chair stand test

ACT, 30-s arm curl test

BST, back scratch test

CSRT, chair sit and reach test

HS, handgrip strength

OLS, one-legged stance

HSPF, handgrip strength and pulmonary function
